# Clinical Scores for Dyspnoea Severity in Children: A Prospective Validation Study

**DOI:** 10.1371/journal.pone.0157724

**Published:** 2016-07-06

**Authors:** Hendriekje Eggink, Paul Brand, Roelien Reimink, Jolita Bekhof

**Affiliations:** 1 Princess Amalia Children’s Clinic, Isala, Zwolle, the Netherlands; 2 University Medical Center Groningen, Groningen, the Netherlands; University Children's Hospital Bern, SWITZERLAND

## Abstract

**Background:**

In acute dyspnoeic children, assessment of dyspnoea severity and treatment response is frequently based on clinical dyspnoea scores. Our study aim was to validate five commonly used paediatric dyspnoea scores.

**Methods:**

Fifty children aged 0–8 years with acute dyspnoea were clinically assessed before and after bronchodilator treatment, a subset of 27 children were videotaped and assessed twice by nine observers. The observers scored clinical signs necessary to calculate the Asthma Score (AS), Asthma Severity Score (ASS), Clinical Asthma Evaluation Score 2 (CAES-2), Pediatric Respiratory Assessment Measure (PRAM) and respiratory rate, accessory muscle use, decreased breath sounds (RAD).

**Results:**

A total of 1120 observations were used to assess fourteen measurement properties within domains of validity, reliability and utility. All five dyspnoea scores showed overall poor results, scoring insufficiently on more than half of the quality criteria for measurement properties. The AS and PRAM were the most valid with good values on six and moderate values on three properties. Poor results were mainly due to insufficient measurement properties in the validity and reliability domains whereas utility properties were moderate to good in all scores.

**Conclusion:**

This study shows that commonly used dyspnoea scores show insufficient validity and reliability to allow for clinical use without caution.

## Introduction

Acute dyspnoea is an important reason for paediatric emergencies and hospital admissions [1]. Assessment of dyspnoea severity and treatment response is essential for therapeutic management. Evaluation of dyspnoea severity in children primarily relies on clinical evaluation, because pulmonary function tests are unreliable and infeasible in acutely dyspnoeic children [2]. This clinical evaluation usually involves a combination of clinical signs, as there is there is no single clinical sign that sufficiently correlates with the degree of dyspnoea or airway narrowing [3–6]. For this purpose, a range of dyspnoea scores, comprising a combination of clinical features and signs, have been developed. Although these are being widely used both in clinical practice and in research, evidence on their measurement properties is limited [7–9]. For instance, adequate information on evaluative quality (responsiveness after treatment) is lacking in all existing scores, limiting their use as evaluative instrument in clinical practice or research. Although the lack of sufficient information from validation studies does not necessarily disqualify existing scores to reliably measure dyspnoea, it does emphasize the need for further validation [7–9].

The aim of this study was to prospectively assess validity and reliability of five commonly used paediatric dyspnoea scores [7–9]: the Asthma Score (AS) [5]; Asthma Severity Score (ASS) [2], Clinical Asthma Evaluation Score 2 (CAES-2) [3], Pediatric Respiratory Assessment Measure (PRAM) [10–11] and the respiratory rate, accessory muscle use, decreased breath sounds (RAD) [12].

## Methods

### Study population and design

We prospectively recruited fifty children aged 0–8 years, presenting with acute dyspnoea and wheeze to the emergency department of Isala, a large teaching hospital in Zwolle, the Netherlands. All children were assessed before and 30 minutes after treatment with a standard dose of nebulized salbutamol (2.5 mg for patients aged < 4 years and 5 mg for ages ≥ 4 years). Only children treated with nebulized salbutamol were included in the study. The emergency department nurse counted the respiratory rate for 1 minute and recorded heart rate and transdermal oxygen saturation by pulse oximetry. If oxygen saturation was <93% or when the child’s dyspnoea was considered to be severe, supplemental oxygen was provided through nasal prongs. Respiratory and heart rate were classified using age-equivalent percentile categories [13]. After bronchodilator administration, the attending clinician rated whether the patient showed improvement, slight improvement, no change or deterioration. In addition, after parental informed consent was obtained to videotape the head and chest of the children before and 30 minutes after bronchodilator administration. The heart rate and transdermal oxygen saturation were visible on the pulse oximetry during the whole recording. Video recording took place in a single-bed room to prevent disturbing noises and ensure the assessors could hear the wheezing, which was confirmed during evaluations by the assessors.

Written parental informed consent was obtained on behalf of the children to videotape the children. The study was approved by the hospital’s medical research and ethics committee Medical Ethical Committee Isala, Zwolle (09.0536n).

### Video assessment

Five paediatricians and four paediatric nurses, each with at least five years of experience in paediatrics, rated the videos. Observers were blinded from clinical information and the timing of the video (before or after bronchodilator treatment). They assessed all necessary items to calculate the five dyspnoea scores: respiratory rate, wheeze, prolonged expiratory phase, retractions (subcostal, intercostal, jugular, supraclavicular), nasal flaring and mental status. Except for respiratory rate, these clinical signs were rated according to a scale ranging from none, mild, moderate to severe. Finally, an overall dyspnoea severity score on a Likert-scale from 0 (no dyspnoea) to 10 (severe dyspnoea defined as respiratory insufficiency) was given. Observers rated all videos twice with an interval of at least two weeks. Participating paediatricians and paediatric nurses gave verbal informed consent.

### Dyspnoea scores

We assessed the dyspnoea scores AS [5], ASS [2], CAES-2 [3], PRAM [10, 11] and RAD [12] (S1 File) because these showed the best measurement properties in a systematic review that we performed earlier on all paediatric dyspnoea scores reported in the literature [9]. Moreover, these five scores were considered suitable for the entire paediatric age span and involved no difficult auscultation skills for the assessment. The range of scores were: AS (5–15), ASS (0–9), CAES-2 (0–8), PRAM (0–12) and RAD (0–3).

We purposely left out auscultatory findings, to reflect daily clinical practice in which healthcare professionals assessing the child are not always trained in pulmonary auscultation, and for reasons of feasibility (having an acutely dyspnoeic child auscultated by nine independent assessors was considered infeasible and excessively distressing to the child).

### Statistical analysis

The five dyspnoea scores were evaluated according to the Consensus-based Standards for the selection of health Measurement Instruments initiative (COSMIN) definitions of fourteen measurement properties for validity, reliability and utility of measures (see S2 File) [14]. Six of these properties (face and content validity, suitability, age span, ease of scoring and auscultation skills) have been described in detail previously [9], and we will explain the methodology used for the other eight measurement properties (construct and criterion-concurrent validity, measurement error, inter and intra-observer reliability, internal consistency, responsiveness and floor and ceiling effects) below.[15]

#### Validity

To assess *construct validity w*e formulated five pre-defined hypotheses about the difference in scores between subgroups in our study sample, three of which referred to evaluative capacity (i.e. the ability to find (small) difference in dyspnoea severity in response to treatment), and two to interpretability (do more children with more severe symptoms have higher scores?). The hypotheses we tested were: 1) Dyspnoea score improvement after treatment with bronchodilator is larger in patients in whom the attending physician observed an improvement after bronchodilator, compared to the stable group; 2) The response to bronchodilator treatment is larger when risk factors for atopy (eczema or a positive family history) are present in comparison to patients without risk factors; 3) Patients diagnosed with episodic wheezing or asthma at discharge show a better response than patients diagnosed with bronchiolitis or pneumonia; 4) Children requiring supplemental oxygen have higher dyspnoea scores than those without oxygen. 5) Change in dyspnoea scores is higher in children who are admitted to the hospital in comparison to children sent home. Sufficient construct validity was reached if 75% of the hypotheses were confirmed by an independent t-test or Mann-Whitney U test.

*Concurrent validity* was evaluated by comparing the total dyspnoea scores with oxygen saturation, age-equivalent respiratory rate percentiles [13] and the 10-point Likert scale for dyspnoea severity. A Pearson or Spearman correlation coefficient of > 0.7 was considered sufficient [14].

#### Reliability

Agreement was quantified by calculating the Smallest Detectable Change (SDC) and Minimal Important Change (MIC) [16]. For a score to be of evaluative value, the SDC must be smaller than the MIC [14]. The SDC was obtained by multiplying the standard deviation of the change in dyspnoea score in the stable group (not importantly improved as judged by the attending physician) by 1.96 [16, 17]. We applied the visual anchor-based MIC distribution to calculate the MIC for each dyspnoea score, by using two external criterions or ‘anchors’ for judgment on the degree of responsiveness to treatment [16]: 1) the clinical judgment of the response to treatment by the paediatrician who assessed the child “live” at the emergency department and 2) the difference in respiratory rate percentile before and after administration of bronchodilator.

To assess *intra- and interrater reliability*, intraclass correlation coefficient (ICC) was calculated (two way mixed models, absolute agreement and single measurements), considering an ICC of ≥0.70 as adequate [13]. Standard error of measurement (SEM) due to variation within observers was calculated by: SEM = SD_difference_/√2 [16]. The SEM due to variation between observers was calculated by using the pooled SD of the mean scores of the different observers using the formula: SEM = SD_pooled_*√(1-ICC). SD_pooled_ was similar to √(SD^2^_observer1_ + SD^2^_observer2_ + …/n) [16, 17]. As the RAD score cannot be regarded a continuous variable with a range of only 4, we chose to use weighted kappa values instead of ICC and did not calculate SEM.

*Internal consistency* was assessed by calculating Cronbach’s alpha [15, 16].

*Responsiveness* was determined calculating the area under curve (AUC) of the receiver operating characteristic curve of the improved versus stable group using the two abovementioned anchors [14]. A value ≥0.70 was considered appropriate.

#### Utility

*Floor or ceiling effects* were evaluated by calculating the percentage of patients with the lowest or highest possible dyspnoea score. Floor and ceiling effects were considered adequate when <15% [16].

All statistical analyses were performed using SPSS version 23.0. P values < 0.05 were considered statistically significant.

## Results

Fifty patients were evaluated before and after administration of bronchodilators, some on several occasions, resulting in 148 observations. Twenty-seven of these patients were videotaped before and after bronchodilator treatment, assessed twice by the nine observers, accounting for 972 video ratings and thus a total of 1120 observations. Patient and clinical characteristics are shown in Table 1.

**Table 1:** Patient characteristics for total group (n = 50) and videotaped participants (n = 27).

	Total (n = 50)		Videotaped children (n = 27)	
Demographics				
Mean age in months (SD)	30.1 (25.8)		24.3 (19.9)	
Male gender, n (%)	30 (60)		17 (63)	
History, n (%)				
Eczema	16 (32)		6 (22)	
Positive family history	40 (80)		23 (85)	
Previous bronchodilators	34 (68)		20 (74)	
Positive effect	19/34 (56)		12 (60)	
Variable/no effect	15/34 (44)		8 (40)	
Clinical characteristics	Pre Tx*	Post Tx*	Pre Tx*	Post Tx*
Respiratory rate				
Breaths/minute, mean (SD)	46 (15)	43 (14)	43 (15)	40 (12)
Tachypnoea^¶^, n (%)	35 (70)	29 (58)	20 (74)	16 (59)
Transdermal oxygen saturation				
%, median (IQR)	96 (92–100)	96 (92–100)	97 (95–98)	97 (94–98)
≤92%, n (%)	7 (14)	4 (8)	7 (26)	4 (15)
Heart rate				
Beats/min, mean (SD)	142 (20)	148 (21)	141 (18)	146 (20)
Tachycardia^¶^, n (%)	31 (62)	33 (66)	18 (67)	19 (70)
Disease course, n (%)				
Hospitalization	34 (68)		19 (70)	
Diagnosis at discharge				
Acute asthma	18 (36)		7 (26)	
Episodic wheeze	20 (40)		13 (48)	
Bronchiolitis/pneumonia	10 (20)		6 (22)	
Other	2 (4)		1 (4)	

SD standard deviation; IQR interquartile range

*before or 30 minutes after treatment with bronchodilators

^¶^ ≥P90 for age according to Fleming et al. [11]

### Validity

The AS, PRAM and RAD showed adequate construct validity, defined by 75% confirmed hypotheses (Table 2). Remarkably, hypothesis 5 showed that the change in dyspnoea scores was rated not rated higher in patients ultimately hospitalised than in the children who were sent home after their visit to the emergency department.

**Table 2:** Construct validity of five dyspnoea scores by testing difference between subgroups in five predefined hypotheses.

1.Physician observation	Improved symptoms after bronchodilators		no changeafter bronchodilators		p-value*	95% CI
AS	-0.65 ± 1.66	(212)	-0.12 ± 1.58	(152)	**0.002**	-0.87–-0.19
ASS	-0.69 ± 1.76	(154)	0.28 ± 1.60	(106)	**0.002**	-1.40–-0.56
CAES-2	-0.70 ± 1.52	(105)	0.08 ± 1.18	(60)	**0.001**	-1.20–-0.35
PRAM	-0.64 ± 2.48	(146)	0.28 ± 2.21	(101)	**0.003**	-1.53–-0.32
RAD	0.00 IQR 1	(146)	0.00 IQR 0	(114)	**0.005**	
2.Risk factors for asthma	Positive risk factors		Negative risk factors			
AS	-0.59 ± 1.72	(452)	0.03 ± 1.87	(75)	**0.004**	-1.04–-0.20
ASS	-0.40 ± 1.77	(336)	-0.08 ± 1.46	(64)	0.127	-0.73–0.09
CAES-2	-0.42 ± 1.43	(216)	-0.34 ± 1.63	(44)	0.741	-0.56–0.40
PRAM	-0.59 ± 2.28	(317)	0.13 ± 2.63	(60)	**0.029**	-1.37–-0.08
RAD	0.00 IQR 1	(329)	0.00 IQR 0	(60)	**0.008**	
3.Diagnosis at discharge	Asthma		Bronchiolitis or pneumonia			
AS	-0.71 ± 1.77	(393)	-0.04 ± 1.28	(95)	**0.001**	-0.98–-0.35
ASS	-0.47 ± 1.78	(316)	0.06 ± 1.49	(53)	**0.044**	-1.03–-0.02
CAES-2	-0.50 ± 1.48	(213)	0.23 ± 1.45	(22)	**0.028**	-1.38–-0.08
PRAM	-0.70 ± 2.35	(299)	0.53 ± 2.19	(51)	**0.001**	-1.92–-0.53
RAD	0.00 IQR 1	(296)	0.00 IQR 0	(66)	**0.034**	
4.Oxygen supplementation	Oxygen supplementation		No oxygen supplementation			
AS	8.76 ± 1.74	(302)	7.34 ± 1.80	(782)	**<0.001**	1.19–1.66
ASS	4.44 ± 1.79	(257)	4.68 ± 1.87	(655)	0.085	-0.32–0.50
CAES-2	1.86 ± 1.69	(204)	1.80 ± 1.33	(492)	0.648	-0.18–0.29
PRAM	4.36 ± 2.19	(116)	2.51 ± 1.97	(331)	**<0.001**	1.42–2.29
RAD	2.00 IQR 0	(245)	2.00 IQR 0	(644)	**0.034**	
5.Hospital admission	Admission		No admission			
AS	-0.54 ± 1.74	(379)	-0.41 ± 1.70	(148)	0.453	-0.46–0.20
ASS	-0.33 ± 1.74	(301)	-0.38 ± 1.70	(99)	0.797	-0.43–0.45
CAES-2	-0.38 ± 1.45	(192)	-0.49 ± 1.51	(68)	0.612	-0.30–0.51
PRAM	-0.61 ± 2.30	(286)	-0.53 ± 2.27	(123)	0.613	-0.39–0.31
RAD	0.00 IQR 0	(548)	0.00 IQR 0	(81)	0.638	

Data is presented as mean ± standard deviation or median with interquartile range (IQR); for the hypothesis 1–3 and 5, change in dyspnoea score after bronchodilator treatment is compared between the two subgroup, and for hypothesis 4 the absolute dyspnoea score is presented. CI confidence interval; AS Asthma score (range 4–12); ASS Asthma severity score (range 0–9); CAES-2 Clinical asthma evaluation score 2 (range 0–8); PRAM Pediatric respiratory assessment measure (range 0–12); RAD Respiratory rate, accessory muscle use, decreased breath sounds (range 0–3)

*T-test for AS, ASS, CAES-2 and PRAM; Mann Whitney U for RAD

Concurrent validity showed an insufficient correlation with the oxygen saturation or respiratory rate percentile for all five scores (Table 3). Correlations with the dyspnoea severity score (Likert scale) were moderate, ranging from 0.441–0.567, and did not exceed the minimum threshold.

**Table 3:** Concurrent validity of five dyspnoea scores by correlating total scores with oxygen saturation, Fleming’s respiratory rate percentile and the dyspnoea severity score.

		Oxygen saturation (%)		Respiratory rate percentile$		Dyspnoea severity score	
		96.0 ± 2.4 (88–100)		7.0 [5–8]		4.2 ± 1.8 (0–8)	
		Pearson’s *r*	*(n)*	Spearman’s *ρ*	*(n)*	Pearson’s *r*	*(n)*
AS	7.74 ± 1.89 (4–12)	-0.324**	(783)	0.132**	(1019)	0.567**	(1019)
ASS	4.61 ± 1.85 (1–8)	-0.096*	(655)	0.161**	(852)	0.543**	(852)
CAES-2	1.82 ± 1.44 (0–8)	-0.021	(492)	0.108**	(648)	0.543**	(648)
PRAM	3.28 ± 2.31 (0–11)	-0.323**	(633)	0.072*	(819)	0.473**	(819)
RAD^#^	2.0 [2–2]	-0.064*	(1059)	0.123**	(994)	0.441**	(994)

Data is presented as mean ± SD (range) or median [interquartile range]; AS Asthma score; ASS Asthma severity score; CAES-2 Clinical asthma evaluation score 2; PRAM Pediatric respiratory assessment measure; RAD Respiratory rate, accessory muscle use, decreased breath sounds;

^$^Age-equivalent percentile categories according to Fleming et al. [11] with 1 (<P1), 2 (P1-10), 3 (P11-25), 4 (P26-50), 5 (P51-75), 6 (P76-90), 7 (P91-99), 8(P>99)

*p<0.05

** p<0.001

^#^ Spearman’s rho correlation coefficient for ordinal RAD score

### Reliability

Results on agreement are presented in Table 4 (for a detailed calculation in S3 File). None of the five dyspnoea scores showed good agreement: in all five the smallest detectable change (SDC) was smaller than the minimal important change (MIC).

**Table 4:** Agreement and responsiveness of the five dyspnoea scores using different anchors of change.

*Anchor*: *‘live’ judgment of clinician on improvement*					
	Difference in dyspnoea score Mean (SD)		SDC	MIC	AUC (95%)
	Improved	Stable			
AS	0.9(1.7)	-0.1 (1.6)	3.12	0 (0.5)	0.65 (0.61–0.70)
ASS	1.2 (1.8)	-0.1 (1.8)	3.53	1 (0.5)	0.70 (0.64–0.75)
CAES-2	1.1 (1.4)	0.1 (1.4)	2.74	0 (0.5)	0.68 (0.61–0.76)
PRAM	1.5 (2.3)	0.0 (2.2)	4.31	0 (0.5)	0.68 (0.62–0.74)
RAD	0.3 (0.7)	-0.1 (0.5)	0.98	0 (0.5)	0.65 (0.61–0.70)
*Anchor*: *change in age-equivalent respiratory rate percentiles*					
	Difference in dyspnoea score Mean (SD)		SDC	MIC	AUC (95%)
	Improved	Stable			
AS	1.0 (1.8)	0.4 (1.7)	3.33	1 (0.5)	0.62 (0.55–0.68)
ASS	0.8 (1.7)	0.04 (1.7)	3.33	0 (0.5)	0.63 (0.57–0.68)
CAES-2	0.9 (1.4)	0.2 (1.4)	2.74	0 (0.5)	0.64 (0.57–0.71)
PRAM	1.1 (2.7)	0.3 (2.2)	4.31	1 (0.5)	0.59 (0.51–0.66)
RAD	0.3 (0.7)	0.07 (0.7)	1.37	0 (0.5)	0.62 (0.57–0.67)

SD standard deviation, SDC smallest detectable change; MIC minimal important change; AUC area under curve; AS Asthma score; ASS Asthma severity score; CAES-2 Clinical asthma evaluation score 2; PRAM Pediatric Respiratory assessment measure; RAD Respiratory rate, accessory muscle use, decreased breath sounds;

Data on intra and interrater reliability and internal consistency are presented in Table 5. Intrarater reliability was good for the AS (ICC 0.75) and ASS (ICC 0.74) and borderline sufficient for the other three scores. Only the AS showed a moderate interrater reliability (ICC 0.64). Internal consistency was inadequate in all scores. For responsiveness, only the ASS reached the threshold of an adequate AUC (Table 4).

**Table 5:** Inter and intrarater reliability and internal consistency of the five dyspnoea scores.

	Overall			Difference in score^¶^		Intrarater reliability		Interrater reliability		Internal consistency
Score	Mean	SD	SD_pool_	Mean	SD_diff_	ICC (95% CI)	SEM	ICC (95% CI)	SEM	Cronbach’s α
AS	7.78	1.91	1.87	0.057	1.36	0.75 (0.71–0.79)	0.96	0.64 (0.56–0.71)	1.12	0.53
ASS	4.61	1.85	1.91	0.018	1.34	0.74 (0.69–0.79)	0.95	0.48 (0.37–0.60)	1.15	0.48
CAES-2	1.82	1.44	1.61	0.022	1.18	0.69 (0.62–0.76)	0.83	0.31 (0.20–0.44)	0.97	0.43
PRAM	3.28	2.31	2.19	0.084	1.91	0.65 (0.59–0.71)	1.35	0.39 (0.27–0.51)	1.31	0.49
						Weighted kappa* (95% CI)		Weighted kappa* (95% CI)		Cronbach’s α
RAD						0.59 (0.51–0.65)		0.32 (0.17–0.53)		0.25

SD standard deviation;SEM Standard Error of Measurement; SD_pool_ pooled SD of the mean scores of the different observers using the formula: √(SD2_observer1_ + SD2_observer2_ + …/n) [16, 17]; SD_diff_ SD of the differences between the two raters [16, 17]; ICC intraclass correlation coefficient; CI confidence interval; AS Asthma score; ASS Asthma severity score; CAES-2 Clinical asthma evaluation score 2; PRAM Pediatric Respiratory assessment measure; RAD Respiratory rate, accessory muscle use, decreased breath sounds

^¶^Difference in the dyspnoea score between the two assessments of the same video by the same rater

*Weighted kappa for ordinal scales, multirater (Light)

### Utility

Floor and ceiling effects were adequate in all five dyspnoea scores (Table 6)

**Table 6:** Floor and ceiling scores and percentages in five dyspnoea scores.

Score	Lowest score	N (%)	Highest score	N (%)	Sum (%)
AS	5	68 (6.1)	12	21 (1.9)	89 (8.0)
ASS	0	0 (0.0)	9	0 (0.0)	0 (0.0)
CAES-2	0	160 (14.3)	8	2 (0.2)	162 (14.5)
PRAM	0	125 (11.2)	12	0 (0.0)	125 (11.5)
RAD	0	21 (1.9)	3	124 (11.1)	145 (13.0)

AS Asthma score; ASS Asthma severity score; CAES-2 Clinical asthma evaluation score 2; RAD Respiratory rate, accessory muscle use, decreased breath sounds

A summary of the assessment of the 5 scores based on our results is given in Table 7.

**Table 7:** Summary of assessed quality criteria for the five dyspnoea scores bases on this study and earlier review by Bekhof et al.[9].

	AS	ASS	CAES-2	PRAM	RAD
*Validity*					
Face	+	+	+	+	+
Content	-	-	-	+	+
Construct	+	-	-	+	+
Concurrent	-	-	-	-	-
*Reliability*					
Agreement	-	-	-	-	-
Inter observer	±	-	-	-	-
Intra observer	+	+	±	±	±
Internal consistency	-	-	-	-	-
Responsiveness	-	±	-	-	-
*Utility*					
Suitability	±	+	+	+	+
Age span	+	+	+	+	-
Ease of scoring	+	±	+	±	+
Auscultation skills	±	±	±	±	±
Floor and ceiling	+	+	+	+	+
Total score	7.5	6.5	6	7.5	7

AS Asthma score; ASS Asthma severity score; CAES-2 Clinical asthma evaluation score 2; RAD Respiratory rate, accessory muscle use, decreased breath sounds;—evaluated and negatively rated, ± evaluated but intermediately positive, + evaluated and positively rated

## Discussion

In this prospective study we aimed to examine external validity of five commonly used dyspnoea scores in children with acute dyspnoea and wheezing. To our knowledge, this is the first study to quantitatively compare measurement properties across dyspnoea scores in dyspnoeic children using independent ratings by nine experienced clinicians. The results show that commonly used dyspnoea scores in children have poor measurement properties, with insufficient results on more than half of the quality criteria for measurement properties (7 to 9 out of 14). The AS and PRAM were evaluated as most valid with good values on six and moderate values on three measurement properties. The poor results were mainly due to insufficient measurement properties in the domains of validity and reliability, whilst all scores performed moderate to well in the domain of utility.

Although assessment of validity (do the scores measure what they intend to do?) is difficult due to the lack of a solid “gold” standard or reference value, all dyspnoea scores scored insufficient on the different aspects of validity which can be assessed in absence of a “gold” standard. None of the composite dyspnoea scores showed good correlations with other single measures of dyspnoea severity, including objective measures (oxygen saturation or respiratory rate) and subjective measures (impression of dyspnoea severity judged by the attending experienced physician). These findings are consistent with earlier reports, showing poor to modest correlation with single clinical signs or arterial oxygen saturation or airway obstruction [3–6]. The AS and PRAM showed a significant higher, but still only slight correlation with oxygen saturation. This is likely to be explained by the fact that SpO2 is an item in these scores, so a higher SpO2 would automatically lead to a lower score on the AS and PRAM. The validity of dyspnoea scores may be hindered by the fact that clinical signs of dyspnoea may vary largely across different ages. Even within the limited age range of preschool children in our study, signs of dyspnoea may differ between young infants and toddlers. A score that is applicable in different settings and across a broad age range is desirable.

The poor validity and especially the poor discriminative and evaluative properties of paediatric dyspnoea scores appears to be mainly due to the large interrater variation. The discriminative power of these composite scores is too low compared to the large variation in the characteristics of the population examined. In other words, the scores do not seem to be sensitive nor precise enough to detect the often subtle changes in clinical conditions in young children. This means that dyspnoea scores should be used with caution. Since none of the scores is significantly better than the others, it would be preferable if clinicians and researchers in the field of paediatric pulmonology could agree on which (selection of) dyspnoea scores are to be used. We would suggest to choose the AS or PRAM since they scored best of all 36 available dyspnoea scores.

Because the large degree of interrater variation limits the validity of these dyspnoea scores in children, efforts to diminish this interrater variation are clearly needed. In the absence of studies examining this, we propose repeated training and discussions over videotaped dyspnoeic patients between the health care professionals. In addition, objective measures to assess severity of dyspnoea in young children are needed. Until such more reliable methods become available, health care professionals caring for acutely dyspnoeic children need to be aware of the unreliability of these scores. This implies that repetitive assessment of dyspnoeic children patients should preferably be done by the same professional, and simultaneous assessment during handovers is warranted.

### Strengths and weaknesses

The main strength of our study is the prospective design, with a sufficient number of patients and observers to enable thorough external validation of different dyspnoea scores simultaneously. This has not been done previously. Furthermore the choice to include patients presenting to the hospital with acute dyspnoea without a specific diagnosis increases the generalizability and applicability of our results, because this patient selection closely reflects daily practice. We used protocolled care for children presenting with acute asthma, pneumonia and bronchiolitis, with clear treatment and admission criteria, although we cannot deny variance in practice between physicians and individualized treatments exist.

The limitations of our study are mainly related to the use of audio-visual recordings and the exclusion of auscultation. The use of videos has its shortcomings in comparison to ‘live’ assessment, because the lack of direct personal contact between patients and caregivers. Nevertheless, video assessment was the most suitable option enabling us to make comparison among multiple clinicians. We left out auscultation because it was difficult to capture with video recordings. This might have led to a score that is different than it would be if auscultation was included. However, previous studies underscored the weak association between auscultatory findings and the actual degree of airway obstruction [2, 18]. Another limitation of our study is that most of our patients were moderately dyspnoeic, not representing the entire range of dyspnoea severity. This may have limited the discriminative power of the scores. However, because severe dyspnoea accounts for only 1–2% of the population we believe that used dyspnoea scores should be especially reliable in moderately dyspnoeic children.

We are aware of the central role of the attending pediatrician in this study. The pediatrician was involved in the clinical decision making with regard to for instance oxygen supplementation and admission to the hospital. However, the aim of the dyspnoea scores is to reflect clinical decision making of clinicians and therefore by comparing the dyspnoea scores to precise these measures seems the most adequate manner to evaluate the applicability of these scores. Furthermore by using several hypotheses without involvement of the attending physician, and by using a second more objective comparison as an anchor (i.e. respiratory rate) we tried to optimize study design and compensate for a lack of a “true gold standard.

## Conclusion

This study is the first to prospectively compare the external validity of five different paediatric dyspnoea scores and enables us to make suggestions about which is the best applicable across the population of dyspnoeic children. We found that all of the scores have poor measurement properties, leading to insufficient validity and reliability. Even the two scores with the best test results (AS and PRAM) lack sufficient discriminative and evaluative power to allow for the sole use as outcome measure for dyspnoea severity in children.

## Supporting Information

S1 File Overview of tested dyspnoea scores. (PDF)

S2 File Quality criteria for measurement properties of paediatric dyspnoea scores. (PDF)

S3 File Calculation of minimal important change for the five dyspnoea scores. (PDF)

S4 File Original data Dyspnoea scores. (XLS)

## Abbreviations

AS: Asthma score
ASS: Asthma severity score
AUC: Area under curve
CAES-2: Clinical asthma evaluation score 2
CI: Confidence interval
COSMIN: Consensus-based standards for the selection of health measurement instruments
ICC: Intraclass correlation coefficient
IQR: Interquartile range
MIC: Minimal important change
PRAM: Pediatric Respiratory assessment measure
RAD: Respiratory rate, accessory muscle use, decreased breath sounds
SD: Standard deviation
SDC: Smallest detectable change
SEM: Standard error of measurements
