# Supplementary material for: Clinical Scores for Dyspnoea Severity in Children: A Prospective Validation Study
Source: PLoS One. 2016 Jul 6;11(7):e0157724. doi: 10.1371/journal.pone.0157724 (PMC4934692; doi:10.1371/journal.pone.0157724)
Supplement: S1 File — (PDF) [file pone.0157724.s001.pdf]

Supplemental file 1: Overview of tested dyspnoea scores

Asthma score (AS) (Qureshi 1998)

Variables	Asthma scoring		
	1 point	2 points	3 points
Respiratory rate (breaths/min)			
2-3yr	≤ 34	35-39	≥ 40
4-5yr	≤ 30	31-35	≥ 36
6-12yr	≤ 26	27-30	≥ 31
>12yr	< 23	24-27	≥ 28
Oxygen saturation (%)	>95 with room air	90-95 with room air	<90 with room air or supplemental oxygen
Auscultation	Normal breathing or end-expiratory wheezing	Expiratory wheezing	Inspiratory and expiratory wheezing, diminished breath sounds or both
Retractions	None or intercostal	Intercostal and substernal	Intercostal, substernal, and supraclavicular
Dyspnea	Speaks in sentences or coos and babbles	Speaks in partial sentences or utters short cries	Speaks single words or short phrases or grunts

Asthma severity score (ASS) (Bishop et al., 1992)

Score	Wheeze	Accessory muscles	Heart rate
0	Absent	0	≤ 80
1	Expiratory only	+	81-110
2	Inspiratory and expiratory	++	111-140
3	Audible without stethoscope or silent chest in severe asthma	+++	≥ 141

Clinical asthma evaluation score (CAES-2) (Hurwitz 1984)

	Score		
Function evaluated	0	1	2
Inspiratory breath sounds	Normal	Unequal	Decreased or absent
Accessory muscle use	None	Moderate (retractions)	Maximal (neck muscles)
Expiratory wheezing	None	Moderate	Marked
Cerebral function	Normal	Depressed or agitated	Coma

Preschool respiratory assessment measure (PRAM) (Chalut et al., 2000; Ducharme et al., 2008)

Signs	0	1	2	3
Suprasternal retractions	Absent		Present	
Scalene muscle contraction	Absent		Present	
Air entry	Normal	Decreased at bases	Widespread decrease	Absent/minimal
Wheezing	Absent	Expiratory only	Inspiratory and expiratory	Audible without stethoscope/silent chest with minimal air entry
O2-saturation	≥ 95%	92-94%	< 92%	

Respiratory rate, accessory muscle use, decreased breath sounds (RAD) (Arnold et al., 2011)

Score component	Operational definition	Scoring
Respiratory rate	Respiratory rate at rest, on air room	≤24 = 0<br>>24 = 1
Accessory muscle use	Any visible use of accessory muscles	Present = 1<br>Not present = 0
Decreased breath sounds	Any decreased breath sounds on auscultation	Normal = 0<br>Any decrease = 1
