# Supplementary material for: Clinical Scores for Dyspnoea Severity in Children: A Prospective Validation Study
Source: PLoS One. 2016 Jul 6;11(7):e0157724. doi: 10.1371/journal.pone.0157724 (PMC4934692; doi:10.1371/journal.pone.0157724)
Supplement: S2 File — (PDF) [file pone.0157724.s002.pdf]

Supplemental file 2: Quality criteria for measurement properties of paediatric dyspnoea scores

Property	Definition	Quality criteria	
**Validity**			
Face validity	*Qualitative judgement if the score is a good measurement of dyspnoea.(Birken 2004)*	+	At least 3 of the following items were part of the score: 1. respiratory and/or heart rate, 2. oxygen saturation or cyanosis, 3. work of breathing, retractions or use of muscles or dyspnoea, 4. wheezing or auscultatory findings, 5. mental status.
		±	2 of the above mentioned items
		-	1 item
Content validity*	*Appropriate representation of the concept dyspnoea by the items in the score (i.e., clear description of development process of the score).(Terwee 2007)*	+	Clear description is provided for measurement aim, target population and item selection and –reduction
		?	Potential methodological shortcomings
		-	No clear description
Construct validity*	*Extent to which the score relates to other measures, consistent with theoretically derived prespecified hypotheses concerning dyspnoea.(Kirschner 1985, Terwee 2007)*	+	Specific hypotheses were formulated and at least 75% of the results are in correspondence with these hypotheses in subgroups of at least 50 patients.
		?	Less than 50 patients OR potential methodological shortcomings or no MIC
		-	Less than 75% of the hypotheses are confirmed
		0	No information
Criterion-concurrent validity*	*Criterion validity refers to the extent to which a score relates to the gold standard of the phenomenon. Because a gold standard of dyspnoea is unavailable, this is replaced by concurrent validity, the degree of agreement with other measurements of dyspnoea.(Terwee 2007)*	+	Valid comparison (oxygen saturation, laboratory findings or pulmonary function tests) and correlation >0,70
		?	Doubts about gold standard
		-	Correlation < 0,70
		0	No information
**Reliability**			
Agreement*	*Absolute measurement error, usually expressed as smallest detectable change (SDC), i.e. the smallest within-person change in score which can be interpreted as real change above measurement error (Terwee 2007)*	+	SDC>MIC or MIC<LOA (Terwee 2007)
		?	Potential methodological shortcomings or no MIC
		-	SDC or LOA ≥ MIC
		0	No information
Inter observer reliability*	*Degree to which different users obtain the same result when using the score on the same patients at the same time*	+	ICC or weighted kappa >0,70 in at least 50 patients
		?	Pearson correlation >0,70, or < 50 patients
		-	ICC or kappa ,0,70
		0	No information
Intra observer reliability*	*Similarity of results when the score is repeated by the same user on the same patient under similar conditions*	+	ICC or weighted kappa >0,70 in at least 50 patients (Terwee 2007)
		?	Pearson correlation coefficient >0,70, or < 50 patients
		-	ICC or kappa < 0,70
		0	No information
Internal consistency*	*Correlation between items of the score (Terwee 2007, streiner 2003)*	+	Factor analysis performed and Cronbach's alfa 0,70-0,95
		?	No factor analysis OR potential methodological shortcomings
		-	Cronbach's alfa < 0,70 or > 0,95
		0	No information
Responsiveness*	*Ability of the score to detect change in time(Guyatt 1992)*	+	Guyatts's RR >1,96 or AUC ≥ 0,70
		?	Potential methodological shortcomings
		-	RR ≤ 1,96 of AUC < 0,70
		0	No information

Utility			
Suitability	*Suitability for use in children*	+	No invasive techniques or items which may be difficult to obtain in young children (e.g. pulsus paradoxus, information on speech not specified for infants).
		±	As in + with information on speech specified for infants
		-	Use of invasive techniques or items which may be difficult to obtain in young children
Age span	*Coverage of the entire paediatric age span*	+	Evaluated from infancy (<2 years) to puberty (>12 years)
		-	Evaluated in a smaller age span
Ease of scoring	*Complexity of scoring system*	+	<4 categories per item (Ducahrme 2008)
		±	4 categories per item
		-	> 4 categories per item or complex calculations needed
Auscultation skills	*Feasibility in clinical practice by different health care providers*	+	no auscultation skills required.
		±	no complex auscultation skills required (no inspiratory:expiratory ratio).
		-	complex auscultation skills required
Floor or ceiling effect*	*Unequal distribution of score results (Terwee 2007)*	+	< 15% of patients with lowest or highest possible score in at least 50 patients
		?	Potential methodological shortcomings or < 50 patients
		-	< 15% of patients with lowest or highest possible score
		0	No information
Interpretability*	*Clinical meaningfulness*	+	Mean scores and SD given in at least 4 relevant subgroups and MIC determined
		?	Potential methodological shortcomings or < 4 subgroups or no MIC determined
		0	No information

+ positive rating; ± indeterminate; - negative rating; ? unclear or potential methodological shortcomings; 0 no information available; (potential) methodological shortcomings = description of design or methods of the study not clear, or study group < 50 persons. *Items together form the Terwee checklist [17]

SDC smallest detectable change; MIC minimal important change; LOA limits of agreement; ICC intraclass correlation coefficient; SD standard deviation; RR Guyatt's responsiveness ratio; AUC area under curve of the receiver operating curve
