# Supplementary material for: Clinical Scores for Dyspnoea Severity in Children: A Prospective Validation Study
Source: PLoS One. 2016 Jul 6;11(7):e0157724. doi: 10.1371/journal.pone.0157724 (PMC4934692; doi:10.1371/journal.pone.0157724)
Supplement: S3 File — (PDF) [file pone.0157724.s003.pdf]

Supplemental file 3: Calculation of minimal important change for the five dyspnoea scores

Table 1A: Distribution of the change in asthma score before and after treatment compared to the predetermined anchors

	Difference in AS before and after treatment with bronchodilators							
		Mean change (SD)	Patients (n)	Observations (n)		Mean change (SD)	Patients (n)	Observations (n)
Clinical assessment	Marked improvement	1.0 (1.6)	19	175	Importantly improved	0.9 (1.7)	35	332
	Slight improvement	0.7 (1.9)	16	157				
	No change	-0.1 (1.6)	15	195	Not importantly improved	-0.1 (1.6)	15	195
Respiratory rate*	4 categories improved	2.0 (2.0)	1	3	Importantly improved	1.0 (1.8)	10	103
	3 categories improved	2.0 (1.3)	2	20				
	2 categories improved	0.7 (1.8)	7	80				
	1 category improved	0.4 (1.4)	7	111	Not importantly improved	0.4 (1.7)	40	424
	No change	0.6 (1.7)	26	248				
	1 category worse	-1.1 (1.6)	4	43				
	2 categories worse	-1	1	1				
	3 categories worse	0.9 (2.0)	1	19				
	5 categories worse	1.5 (0.0-3.0)	1	2				

Table 1B: Determination of the MIC by the ROC-curve method for the 'live clinical assessment' anchor

Change in AS	Importantly improved		Not importantly improved		ROC cut-off	Sensitivity	(1-sens)	Specificity	(1-spec)	(1-sens) + (1-spec)
	(n)	(%)	N	(%)						
-5	0	0	1	0.5	-5	1	0	0.00512821	0.99487179	0.99487179
-4	2	0.6	3	1.5	-4.5	0.9939759	0.0060241	0.02051282	0.97948718	0.98551128
-3	6	1.8	10	5.1	-3.5	0.97590361	0.0240964	0.07179487	0.92820513	0.95230151
-2	17	5.1	19	9.7	-2.5	0.9246988	0.0753012	0.16923077	0.83076923	0.90607044
-1	48	14.5	37	19	-1.5	0.78012048	0.2198795	0.35897436	0.64102564	0.86090516
**0**	**61**	**18.4**	**57**	**29.2**	**-0.5**	**0.5963855**	**0.403614**	**0.6512821**	**0.3487179**	**0.7523324**
1	89	26.8	45	23.1	0.5	0.32831325	0.6716867	0.88205128	0.11794872	0.78963546
2	56	16.9	14	7.2	1.5	0.15963855	0.8403614	0.95384615	0.04615385	0.88651529
3	30	9	4	2.1	2.5	0.06927711	0.9307229	0.97435897	0.02564103	0.95636392
4	17	5.1	5	2.6	3.5	0.01807229	0.9819277	1	0	0.98192771
5	5	1.5	0	0	4.5	0.00301205	0.996988	1	0	0.99698795
6	1	0.3	0	0	6	0	1	1	0	1

Table 1C: Determination of the MIC by the ROC-curve method for the 'change in respiratory rate in percentiles' anchor

Change in AS	Importantly improved		Not importantly improved		ROC cut-off	Sensitivity	(1-sens)	Specificity	(1-spec)	(1-sens) + (1-spec)
	(n)	(%)	N	(%)						
-5	0	0	1	0.2	-5	1	0	0.00235849	0.99764151	0.99764151
-4	1	1	4	0.9	-4.5	0.99029126	0.0097087	0.01179245	0.98820755	0.99791629
-3	2	1.9	14	3.3	-3.5	0.97087379	0.0291262	0.04481132	0.95518868	0.98431489
-2	8	7.8	28	6.6	-2.5	0.89320388	0.1067961	0.11084906	0.88915094	0.99594706
-1	7	6.8	78	18.4	-1.5	0.82524272	0.1747573	0.29481132	0.70518868	0.87994596
0	18	17.5	100	23.6	-0.5	0.65048544	0.3495146	0.53066038	0.46933962	0.81885419
**1**	**26**	**25.2**	**108**	**25.5**	**0.5**	**0.3980583**	**0.601942**	**0.7853774**	**0.2146226**	**0.8165644**
2	20	19.4	50	11.8	1.5	0.2038835	0.7961165	0.90330189	0.09669811	0.89281462
3	14	13.6	20	4.7	2.5	0.06796117	0.9320388	0.9504717	0.0495283	0.98156714
4	5	4.9	17	4	3.5	0.01941748	0.9805825	0.99056604	0.00943396	0.99001649
5	2	1.9	3	0.7	4.5	0	1	0.99764151	0.00235849	1.00235849
6	0	0	1	0.2	6	0	1	1	0	1

Table 2A: Distribution of the change in asthma severity score before and after treatment compared to the predetermined anchors

Difference in ASS before and after treatment with bronchodilators								
		Mean change (SD)	Patients (n)	Observations (n)		Mean change (SD)	Patients (n)	Observations (n)
Clinical assessment	Marked improvement	1.2 (1.8)	19	135	Importantly improved	1.2 (1.8)	19	135
	Slight improvement	0.3 (1.5)	16	119	Not importantly improved	-0.1 (1.8)	31	265
	No change	-0.4 (1.6)	15	146				
Respiratory rate*	4 categories improved	0.0 (0.0)	1	2	Importantly improved	0.8 (1.7)	18	161
	3 categories improved	1.4 (1.0)	2	20				
	2 categories improved	0.9 (1.9)	7	61				
	1 category improved	0.6 (1.7)	7	78				
	No change	0.1 (1.8)	26	185	Not importantly improved	0.04 (1.7)	32	239
	1 category worse	0.0 (1.5)	4	35				
	3 categories worse	-0.7 (1.1)	1	17				
	5 categories worse	0.0 (-1.0-1.0)	1	2				

Table 2B: Determination of the MIC by the ROC-curve method for the 'live clinical assessment' anchor

Change in ASS	Importantly improved		Not importantly improved		ROC cut-off	Sensitivity	(1-sens)	Specificity	(1-spec)	(1-sens) + (1-spec)
	(n)	(%)	N	(%)						
-4	0	0	4	1.5	-4	1	0	0.01509434	0.98490566	0.98490566
-3	1	0.7	12	4.5	-3.5	0.99259259	0.0074074	0.06037736	0.93962264	0.94703005
-2	6	4.4	29	10.9	-2.5	0.94814815	0.0518519	0.16981132	0.83018868	0.88204053
-1	13	9.6	55	20.8	-1.5	0.85185185	0.1481481	0.37735849	0.62264151	0.77078966
0	34	25.2	74	27.9	-0.5	0.6	0.4	0.65660377	0.34339623	0.74339623
**1**	**28**	**20.7**	**56**	**21.1**	**0.5**	**0.3925926**	**0.607407**	**0.8679245**	**0.1320755**	**0.7394829**
2	22	16.3	19	7.2	1.5	0.22962963	0.7703704	0.93962264	0.06037736	0.83074773
3	16	11.9	13	4.9	2.5	0.11111111	0.8888889	0.98867925	0.01132075	0.90020964
4	9	6.7	3	1.1	3.5	0.04444444	0.9555556	1	0	0.95555556
5	6	4.4	0	0	5	0	1	1	0	1

Table 2C: Determination of the MIC by the ROC-curve method for the 'change in respiratory rate in percentiles' anchor

Change in ASS	Importantly improved		Not importantly improved		ROC cut-off	Sensitivity	(1-sens)	Specificity	(1-spec)	(1-sens) + (1-spec)
	(n)	(%)	N	(%)						
-4	0	0	4	1.7	-4	1	0	0.0167364	0.9832636	0.9832636
-3	3	1.9	10	4.2	-3.5	0.98136646	0.0186335	0.05857741	0.94142259	0.96005613
-2	10	6.2	25	10.5	-2.5	0.91925466	0.0807453	0.16317992	0.83682008	0.91756543
-1	21	13	47	19.7	-1.5	0.78881988	0.2111801	0.35983264	0.64016736	0.85134749
**0**	**40**	**24.8**	**68**	**28.5**	**-0.5**	**0.5403727**	**0.459627**	**0.6443515**	**0.3556485**	**0.8152759**
1	36	22.4	48	20.1	0.5	0.31677019	0.6832298	0.84518828	0.15481172	0.83804153
2	24	14.9	17	7.1	1.5	0.16770186	0.8322981	0.91631799	0.08368201	0.91598015
3	17	10.6	12	5	2.5	0.0621118	0.9378882	0.9665272	0.0334728	0.971361
4	6	3.7	6	2.5	3.5	0.02484472	0.9751553	0.9916318	0.0083682	0.98352348
5	4	2.5	2	0.8	5	0	1	1	0	1

Table 3A: Distribution of the change in CAES-2 before and after treatment compared to the predetermined anchors

Difference in CAES-2 before and after treatment with bronchodilators								
		Mean change (SD)	Patients (n)	Observations (n)		Mean change (SD)	Patients (n)	Observations (n)
Clinical assessment	Marked improvement	1.1 (1.4)	19	81	Importantly improved	1.1 (1.4)	19	81
	Slight improvement	0.4 (1.4)	16	86	Not importantly improved	0.1 (1.4)	31	179
	No change	-0.2 (1.3)	15	93				
Respiratory rate*	3 categories improved	1.3 (0.9)	2	16	Importantly improved	0.9 (1.4)	16	85
	2 categories improved	0.7 (1.8)	7	80				
	1 category improved	0.5 (1.7)	7	37				
	No change	0.2 (1.5)	26	136	Not importantly improved	0.2 (1.4)	34	175
	1 category worse	-0.1 (1.1)	4	20				
	2 categories worse	0.0 (0.0)	1	1				
	3 categories worse	0.3 (1.2)	1	16				
	5 categories worse	1.5 (0.0-3.0)	1	2				

Table 3B: Determination of the MIC by the ROC-curve method for the 'live clinical assessment' anchor

Change in CAES-2	Importantly improved		Not importantly improved		ROC cut-off	Sensitivity	(1-sens)	Specificity	(1-spec)	(1-sens) + (1-spec)
	(n)	(%)	N	(%)						
-5	0	0	1	0.6	-5	1	0	0.00558659	0.99441341	0.99441341
-4	0	0	2	1.1	-4.5	1	0	0.01675978	0.98324022	0.98324022
-3	0	0	2	1.1	-3.5	1	0	0.02793296	0.97206704	0.97206704
-2	3	3.7	20	11.2	-2.5	0.96296296	0.037037	0.1396648	0.8603352	0.89737223
-1	5	6.2	12	6.7	-1.5	0.90123457	0.0987654	0.20670391	0.79329609	0.89206152
**0**	**22**	**27.2**	**86**	**48**	**-0.5**	**0.6296296**	**0.37037**	**0.6871508**	**0.3128492**	**0.6832195**
1	20	24.7	29	16.2	0.5	0.38271605	0.617284	0.84916201	0.15083799	0.76812194
2	20	24.7	22	12.3	1.5	0.13580247	0.8641975	0.97206704	0.02793296	0.89213049
3	7	8.6	4	2.2	2.5	0.04938272	0.9506173	0.99441341	0.00558659	0.95620388
4	2	2.5	1	0.6	3.5	0.02469136	0.9753086	1	0	0.97530864
5	2	2.5	0	0	5	0.00301205	0.996988	1	0	0.99698795

Table 3C: Determination of the MIC by the ROC-curve method for the 'change in respiratory rate in percentiles' anchor

Change in CAES-2	Importantly improved		Not importantly improved		ROC cut-off	Sensitivity	(1-sens)	Specificity	(1-spec)	(1-sens) + (1-spec)
	(n)	(%)	N	(%)						
-5	0	0	1	0.6	-5	1	0	0.00571429	0.99428571	0.99428571
-4	1	1.2	1	0.6	-4.5	0.98823529	0.0117647	0.01142857	0.98857143	1.00033613
-3	0	0	2	1.1	-3.5	0.98823529	0.0117647	0.02285714	0.97714286	0.98890756
-2	3	3.5	20	11.4	-2.5	0.95294118	0.0470588	0.13714286	0.86285714	0.90991597
-1	3	3.5	14	8	-1.5	0.91764706	0.0823529	0.21714286	0.78285714	0.86521008
**0**	**29**	**34.1**	**79**	**45.1**	**-0.5**	**0.5764706**	**0.423529**	**0.6685714**	**0.3314286**	**0.754958**
1	24	28.2	25	14.3	0.5	0.29411765	0.7058824	0.81142857	0.18857143	0.89445378
2	16	18.8	26	14.9	1.5	0.10588235	0.8941176	0.96	0.04	0.93411765
3	7	8.2	4	2.3	2.5	0.02352941	0.9764706	0.98285714	0.01714286	0.99361345
4	0	0	3	1.7	3.5	0.02352941	0.9764706	1	0	0.97647059
5	2	2.4	0	0	5	0	1	1	0	1

Table 4A: Distribution of the change in PRAM score before and after treatment compared to the predetermined anchors

	Difference in PRAM before and after treatment with bronchodilators							
		Mean change (SD)	Patients (n)	Observations (n)		Mean change (SD)	Patients (n)	Observations (n)
Clinical assessment	Marked improvement	1.5 (2.3)	19	126	Importantly improved	1.5 (2.3)	19	126
	Slight improvement	0.4 (2.3)	16	113	Not importantly improved	0.0 (2.2)	31	251
	No change	-0.4 (2.1)	15	138				
Respiratory rate*	4 categories improved	4.0 (2.0-6.0)	1	2				
	3 categories improved	1.1 (1.8)	2	17	Importantly improved	1.1 (2.7)	10	80
	2 categories improved	1.0 (2.9)	7	61				
	1 category improved	0.3 (1.9)	7	69				
	No change	0.41 (2.4)	26	177				
	1 category worse	-1.1 (1.6)	4	32	Not importantly improved	0.3 (2.2)	40	297
	2 categories worse	0.0 (0.0)	1	1				
	3 categories worse	0.4 (1.7)	1	16				
	5 categories worse	2.0 (0.0-4.0)	1	2				

Table 4B: Determination of the MIC by the ROC-curve method for the 'live clinical assessment' anchor

Change in PRAM	Importantly improved		Not importantly improved		ROC cut-off	Sensitivity	(1-sens)	Specificity	(1-spec)	(1-sens) + (1-spec)
	(n)	(%)	N	(%)						
-7	0	0	1	0.4	-7	1	0	0.00398406	0.99601594	0.99601594
-6	0	0	0	0	-6.5	1	0	0.00398406	0.99601594	0.99601594
-5	0	0	2	0.8	-5.5	1	0	0.01195219	0.98804781	0.98804781
-4	3	2.4	16	6.4	-4.5	0.97619048	0.0238095	0.07569721	0.92430279	0.94811231
-3	1	0.8	6	2.4	-3.5	0.96825397	0.031746	0.09960159	0.90039841	0.93214444
-2	6	4.8	37	14.7	-2.5	0.92063492	0.0793651	0.24701195	0.75298805	0.83235313
-1	11	8.7	26	10.4	-1.5	0.83333333	0.1666667	0.35059761	0.64940239	0.81606906
**0**	**28**	**22.2**	**82**	**32.7**	**-0.5**	**0.6111111**	**0.388889**	**0.6772908**	**0.3227092**	**0.7115981**
1	12	9.5	18	7.2	0.5	0.51587302	0.484127	0.74900398	0.25099602	0.735123
2	33	26.2	41	16.3	1.5	0.25396825	0.7460317	0.9123506	0.0876494	0.83368115
3	9	7.1	6	2.4	2.5	0.18253968	0.8174603	0.93625498	0.06374502	0.88120534
4	12	9.5	9	3.6	3.5	0.08730159	0.9126984	0.97211155	0.02788845	0.94058686
5	5	4	3	1.2	4.5	0.04761905	0.952381	0.98406375	0.01593625	0.96831721
6	2	1.6	3	1.2	5.5	0.03174603	0.968254	0.99601594	0.00398406	0.97223803

7	2	1.6	0	0	6.5	0.01587302	0.984127	0.99601594	0.00398406	0.98811105
8	1	0.8	1	0.4	7.5	0.00793651	0.9920635	1	0	0.99206349
9	1	0.8	0	0	9	0	1	1	0	1

Table 4C: Determination of the MIC by the ROC-curve method for the 'change in respiratory rate in percentiles' anchor

Change in PRAM	Importantly improved		Not importantly improved		ROC cut-off	Sensitivity	(1-sens)	Specificity	(1-spec)	(1-sens) + (1-spec)
	(n)	(%)	N	(%)						
-7	0	0	0	0.3	-7	1	0	0	1	1
-6	0	0	0	0	-6.5	1	0	0	1	1
-5	0	0	2	0.7	-5.5	1	0	0.00675676	0.99324324	0.99324324
-4	5	6.3	14	4.7	-4.5	0.9375	0.0625	0.05405405	0.94594595	1.00844595
-3	2	2.5	5	1.7	-3.5	0.9125	0.0875	0.07094595	0.92905405	1.01655405
-2	6	7.5	37	12.5	-2.5	0.8375	0.1625	0.19594595	0.80405405	0.96655405
-1	5	6.3	32	10.8	-1.5	0.775	0.225	0.30405405	0.69594595	0.92094595
0	20	25	90	30	-0.5	0.525	0.475	0.60810811	0.39189189	0.86689189
**1**	**4**	**5**	**26**	**8.8**	**0.5**	**0.475**	**0.525**	**0.6959459**	**0.3040541**	**0.8290541**
2	21	26.3	53	17.8	1.5	0.2125	0.7875	0.875	0.125	0.9125
3	3	3.8	12	4	2.5	0.175	0.825	0.91554054	0.08445946	0.90945946
4	7	8.8	14	4.7	3.5	0.0875	0.9125	0.96283784	0.03716216	0.94966216
5	1	1.3	7	2.4	4.5	0.075	0.925	0.98648649	0.01351351	0.93851351
6	3	3.8	2	0.7	5.5	0.0375	0.9625	0.99324324	0.00675676	0.96925676
7	1	1.3	1	0.3	6.5	0.025	0.975	0.99662162	0.00337838	0.97837838
8	1	1.3	1	0.3	7.5	0.0125	0.9875	1	0	0.9875
9	1	1.3	0	0	9	0	1	1	0	1

Table 5A: Distribution of the change in RAD score before and after treatment compared to the predetermined anchors

	Difference in RAD before and after treatment with bronchodilators							
		Mean change (SD)	Patients (n)	Observations (n)		Mean change (SD)	Patients (n)	Observations (n)
Clinical assessment	Marked improvement	0.4 (0.7)	19	169	Importantly improved	0.3 (0.7)	35	318
	Slight improvement	0.2 (0.5)	16	149				
	No change	-0.1 (0.5)	15	188	Not importantly improved	-0.1	15	188
Respiratory rate*	4 categories improved	0.7 (0.0-1.0)	1	3	Importantly improved	0.3 (0.7)	18	204
	3 categories improved	1.0 (0.8)	2	18				
	2 categories improved	0.1 (0.6)	7	78				
	1 category improved	0.3 (0.7)	7	105				
	No change	0.1 (0.7)	26	240	Not importantly improved	0.07 (0.7)	32	302
	1 category worse	-0.1 (0.5)	4	41				
	3 categories worse	-0.1 (0.3)	1	18				
	5 categories worse	0.0 (-1.0-1.0)	1	2				

Table 5B: Determination of the MIC by the ROC-curve method for the 'live clinical assessment' anchor

Change in RAD	Importantly improved		Not importantly improved		ROC cut-off	Sensitivity	(1-sens)	Specificity	(1-spec)	(1-sens) + (1-spec)
	N	(%)	N	(%)						
-2	4	1.3	2	1.1	-2	0.98742138	0.012579	0.01069519	0.98930481	1.00188343
-1	21	6.6	25	13.8	-1.5	0.92138365	0.078616	0.14438503	0.85561497	0.93423133
**0**	**195**	**61.3**	**141**	**75**	**-0.5**	**0.3081761**	**0.691824**	**0.89839572**	**0.10160428**	**0.79342818**
1	80	25.2	18	9.6	0.5	0.05660377	0.943396	0.99465241	0.00534759	0.94874382
2	17	5.3	1	0.5	1.5	0.00314465	0.996855	1	0	0.99685535
3	1	0.3	0	0	3	0	1	1	0	1

Table 5C: Determination of the MIC by the ROC-curve method for the 'change in respiratory rate in percentiles' anchor

Change in RAD	Importantly improved		Not importantly improved		ROC cut-off	Sensitivity	(1-sens)	Specificity	(1-spec)	(1-sens) + (1-spec)
	N	(%)	N	(%)						
-2	0	0	6	2	-2	1	0	0.01986755	0.98013245	0.98013245
-1	15	6.4	32	10.6	-1.5	0.92647059	0.073529	0.12582781	0.87417219	0.9477016
**0**	**130**	**63.7**	**206**	**68.2**	**-0.5**	0.28921569	**0.710784**	0.80794702	**0.19205298**	**0.90283729**
1	46	22.5	52	17.2	0.5	0.06372549	0.936275	0.98013245	0.01986755	0.95614206
2	12	5.9	6	2	1.5	0.00490196	0.995098	1	0	0.99509804
3	1	0.5	0	0	3	0	1	1	0	1
